# Hypoxia-mediated autophagic flux inhibits silver nanoparticle-triggered apoptosis in human lung cancer cells

**DOI:** 10.1038/srep21688

**Published:** 2016-02-12

**Authors:** Jae-Kyo Jeong, Sangiliyandi Gurunathan, Min-Hee Kang, Jae Woong Han, Joydeep Das, Yun-Jung Choi, Deug-Nam Kwon, Ssang-Goo Cho, Chankyu Park, Han Geuk Seo, Hyuk Song, Jin-Hoi Kim

**Affiliations:** 1 Department of Animal Biotechnology, Konkuk University, Seoul 143-701, Republic of Korea

## Abstract

Solid tumors are frequently associated with resistance to chemotherapy because the fraction of hypoxic tumor cells is substantial. To understand the underlying mechanism of hypoxia on silver nanoparticle (AgNPs)-induced apoptosis, the expression of hypoxia-inducible factor (HIF)-1α, a hallmark of hypoxia, was measured in the presence and absence of AgNPs. The results showed that HIF-1α expression was upregulated after AgNPs treatment under both hypoxic and normoxic conditions. Cell viability assays showed that AgNPs promoted cell death in cancer cells but not in non-cancer cells, as cancer cells are slightly more acidic than normal cells. However, reactive oxygen species generation induced by AgNPs in lung cancer cells caused high susceptibility to oxidative stress, whereas pre-exposure to hypoxia blocked AgNPs-induced oxidative stress. Notably, HIF-1α inhibited AgNPs-induced mitochondria-mediated apoptosis by regulating autophagic flux through the regulation of ATG5, LC3-II, and p62. Further, cell viability after treatment of cancer cells with AgNPs under hypoxic conditions was lower in HIF-1α siRNA-transfected cells than in control siRNA-transfected cells, indicating that HIF-1α knockdown enhances hypoxia induced decrease in cell viability. Our results suggest that hypoxia-mediated autophagy may be a mechanism for the resistance of AgNPs-induced apoptosis and that strategies targeting HIF-1α may be used for cancer therapy.

Silver nanoparticles (AgNPs) are of industrial, academic, and scientific interest because of their unique physical, chemical, optical, catalytic, and antibacterial properties^1^. AgNPs have been used in many different applications and in a wide range of products, such as wound dressing, and for coating work surfaces, surgical instruments, and prostheses^1^. Furthermore, AgNPs have been extensively used as antibacterial, antifungal, antiviral, anti-inflammatory, and anti-angiogenic agents. Because of the dramatic expansion of the nanotechnology industry and the increase in the use of nanomaterials, investigation into the potential toxic effects of nanoparticles (NPs) on human health is essential^1^,^2^.

A number of studies have demonstrated associations between AgNPs-mediated cytotoxicity, oxidative stress, and apoptosis^3^,^4^. AgNPs can bind to cells and activate cellular signaling processes that promote reactive oxygen species (ROS) production, inflammation, and finally cell cycle arrest or cell death^3^,^5^. Lee *et al*.^6^ showed that AgNPs treatment impaired cellular proliferation and mitochondrial function, induced apoptosis, and decreased autophagic flux in NIH 3T3 cells. Several *in vivo* studies have also revealed increased level of ROS in the sera of AgNP-treated rats^7^ and up-regulation of oxidative stress-related genes in the caudate nucleus, frontal cortex, and hippocampus of AgNP-treated mice^8^. Herzog *et al*. investigated whether silver ions reflect physiological exposure conditions in human lung cells and concluded that the AgNPs do not cause adverse effects and that the cells were only sensitive to high Ag-ion concentrations^9^.

Previous studies have suggested AgNPs have toxic effects in various cell types, including in prokaryotic and eukaryotic cells, and that AgNPs may be used in oncologic therapy^10^. Moreover, Sriram *et al*.^11^ demonstrated the efficacy of biologically synthesized AgNPs as antitumor agents by using Dalton’s lymphoma ascites cell lines *in vitro* and *in vivo*.

Hypoxia, a condition of decreased oxygen availability, contributes to pathophysiology in various cells and tissues. However, to cope with hypoxic stress, cells possess a finely tuned regulatory system involving hypoxia-inducible transcription factors^12^,^13^. Upon activation, hypoxia-inducible factor (HIF)-1 binds to hypoxia-responsive elements in specific genes to induce their expression. The expression of these genes promotes oxygen delivery, enhances the cellular capacity for anaerobic metabolism, or protects cells from injury and death. Hypoxia decreases the effects of anticancer drugs in solid tumor cells through the regulation of HIF-1α^14^. Composed of α- and β-subunits, HIF-1α is a key regulator of the metabolic adaptation to hypoxia^15^. Under hypoxic conditions, HIF-1α activates multiple target genes^15^. HIF-1α has been implicated as an oncogene that is overexpressed in some human cancer cells. Blockade of HIF-1α, alone or in combination with chemotherapeutic reagents, has been investigated as a therapeutic target^16^. HIF-1α has also been implicated as a co-regulator of autophagy activation to regulate apoptosis^17^. While mild or moderate hypoxia can promote cell viability by activating survival genes, prolonged and severe hypoxia induces cell death, which is associated with the regulation of autophagic flux^18^,^19^. Considering that HIF-1α regulates the expression of both pro-survival and cell death-inducing genes^20^,^21^, it is crucial to understand how fine-tuning of this regulatory mechanism determines life and death.

Autophagy is a mechanism for the degradation of organelles and protein aggregates through the lysosomal pathway^22^. As such, autophagy plays a pivotal role in anticancer and neuroprotective mechanisms^23^,^24^. In particular, hypoxia-induced autophagy inhibits chemotherapeutic effects through a cytoprotective adaptive response, thereby promoting the resistance to treatment^25^. Human hepatoma cell lines undergo acute cell death under starvation conditions, but hypoxia allows the cells to survive under these conditions^26^. Exposure of cells and tissues to hypoxic conditions causes vasodilatation, angiogenesis, erythropoiesis, and glycolysis^27^, although these responses do not provide a sufficient supply of nutrients to the cells. For cellular survival in a hypoxic and nutrient-deprived microenvironment, other metabolic processes, such as autophagy, can be activated to provide energy. Autophagy is a catabolic process that enables cells to recycle amino acids and other intracellular nutrients to obtain energy. The process is evolutionarily conserved in eukaryotes from yeast to mammals^28^. Autophagy is triggered by the formation of autophagosomes, which requires the microtubule-associated protein 1A/1B-light chain 3 (LC3) protein and protein-protein conjugation systems^29^,^30^. Next, autophagosomes fused to the lysosome form the autolysosome to degrade its load via the interaction between LC3 and p62 (ubiquitin-binding protein)^31^,^32^. The LC3/p62 conjugate is also degraded in the autolysosomes^33^,^34^,^35^. Additionally, it is well known that autophagy protect cells under abnormal conditions including starvation, stroke, and hypoxic condition^36^,^37^. Noman *et al*. reported a novel functional link between hypoxia-induced autophagy and the regulation of antigen-specific T-cell lysis and a major role of autophagy in the control of *in vivo* tumor growth^38^. Although autophagy has been studied extensively, the protective role of autophagic flux induced by hypoxia in AgNPs-induced apoptosis has not been well characterized.

The first aim of this study was to investigate the toxicity of biologically synthesized AgNPs in lung cancer cells. The second aim was to examine the effect of hypoxia on AgNPs-induced apoptosis. The final aim was to understand the roles of hypoxia and autophagy in the resistance of cancer cells to AgNPs-induced cell death and also to understand the mechanisms that inhibit apoptosis or promote cell survival under hypoxia. For this purpose, human alveolar basal epithelial cell lines (A549 and L132) were exposed to AgNPs, which are among the most important nanoparticles used in cancer therapy. The reason for chosen of A549 cells, which are known to express higher levels of more stable HIF-1α, which is important for tumor cells with limited oxygen supplies and it, is involved in proliferation and angiogenesis.

## Results

### Characterization of AgNPs and effects of hypoxia on cell death

**Figure 1:**
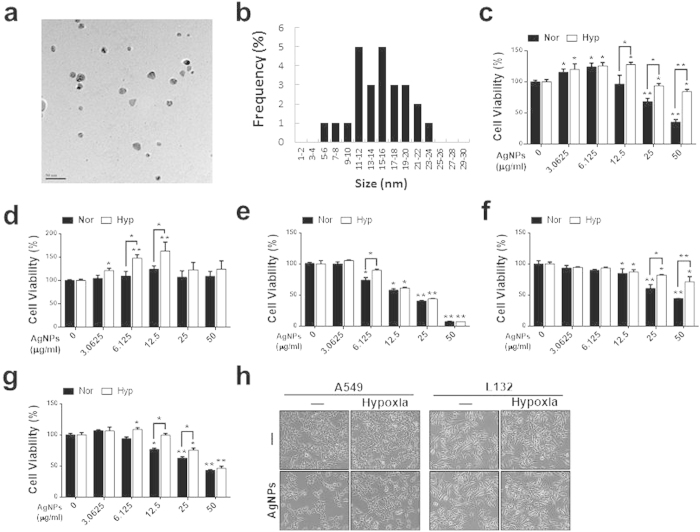
Determination of AgNPs size and the dose-dependent effect of AgNPs on A549 and L132 cells. The size and morphology of AgNPs were determined using TEM. (**a**) TEM micrograph of AgNPs prepared from *Bacillus flexus.* The average particle size was found to be 10 nm. (**b**) Particle size distributions from TEM images. **(c**) A549 (**d**) L132 (**e**) A2780, (**f**) MCF-7, and (**g**) MDA-MB 231 cell lines were incubated with different concentrations of AgNPs with or without 12 h hypoxia pre-exposure. The bar graph indicates the mean ± SEM (*n* = 3). *P < 0.05, **P < 0.01, significant differences between control and each treatment group. (**h**) Treated cells were photographed under a light microscope (200×).

AgNPs were synthesized using *Bacillus flexus*, and the synthesized NPs were primarily characterized using UV–visible spectroscopy. In the UV–visible spectrum, a strong, broad peak, located at approximately 420 nm, was observed for AgNPs prepared using the biological system. The sizes of synthesized NPs were analyzed using transmission electron microscopy (TEM), which demonstrated that the particle size ranged from 10 to 20 nm, with an average size of 10 nm (Fig. 1a,b).

We examined the effect of hypoxia on AgNPs-induced cell death in A549 (lung epithelial cancer cells) and L132 (normal lung epithelial cells), human ovarian cancer cells (2780), human breast cancer cells such as MCF-7 and MDA-MB 231 cells by assessing changes in cell viability. A549, 2780, MCF-7 and MDA-MB 231 cells were treated with various concentrations (0, 3.0625, 6.125, 12.5, 25, 50 μg/mL) of AgNPs alone or after exposure to hypoxic conditions for 12 h. A dose-dependent decrease in cell viability was observed in tested cancer cells, which were treated with AgNPs for 24 h, whereas there was no significant difference in the viability of untreated and AgNPs-treated L132 cells. Cell viability studies involving AgNPs were carried out over the concentration range of 0–50 μg/mL. The results suggest that all type of cancer cells showed similar trend of cell viability (Fig. 1c–g). However, we selected A549 cells for further study, because it is well known that A549 cells expressing higher levels of more stable HIF-1α, which is important for tumor cells with limited oxygen supplies and it, is involved in proliferation and angiogenesis. To narrow down further studies, we determined the IC50 value of AgNPs in A549 cells.

The results suggested that 32.33 μg/mL AgNPs decreased the viability of A549 cells to 50% of the control level, and thus this was determined to be the IC_50_. Exposure to higher concentrations resulted in increased toxicity to the cells (Fig. 1c,e–g)). Moreover, the apoptosis assays showed that hypoxic exposure inhibited the chemotherapeutic effects of AgNPs in A549 lung cancer cells, but had no effect on L132 normal lung cells (Fig. 1d). As shown in Fig. 1h (left) A549 cells treated with AgNPs were rounded and detached from the plate. Under hypoxia conditions, the AgNPs-induced morphological changes were inhibited. In contrast, hypoxia had no effect on AgNPs-induced morphological changes in L132 normal lung cells (Fig. 1h, Right). Collectively, these results are consistent with the hypothesis that hypoxia decreases AgNPs-mediated, cancer cell-specific death/apoptosis. Therefore, further experiments were carried out using only A549 lung cancer cells.

### Cellular uptake of AgNPs caused the accumulation of autophagosomes and autolysosomes

TEM analysis of A549 untreated cells revealed a clear nuclear morphology without damage to any other organelles (Supplementary Fig. 1a), whereas AgNPs-treated cells showed AgNPs localized on or around autophagosomal membranes (Supplementary Fig. 1b–1f), likely because of the loss of membrane integrity. In contrast to untreated cells, AgNPs-treated cells contained several multivesicular and membrane-rich autophagosomes in close proximity to one other, indicating that AgNPs induce autophagosome formation, alter cellular homeostasis, and alter the adaptation to stress in A549 cells. Furthermore, ultrastructural observations showed that A549 cells exposed to AgNPs contained double-membrane autophagosomes with typical cellular contents and an autolysosome. Furthermore, AgNPs-treated cells showed numerous vesicles (autophagosomes), damaged mitochondria, and autolysosome, in the cytoplasm (Supplementary Fig. 1g–i). In contrast, no significant difference was observed in normal lung cancer cells (data not shown).

### Hypoxia inhibited AgNPs-induced, mitochondria-mediated damage by regulating oxidative stress

**Figure 2:**
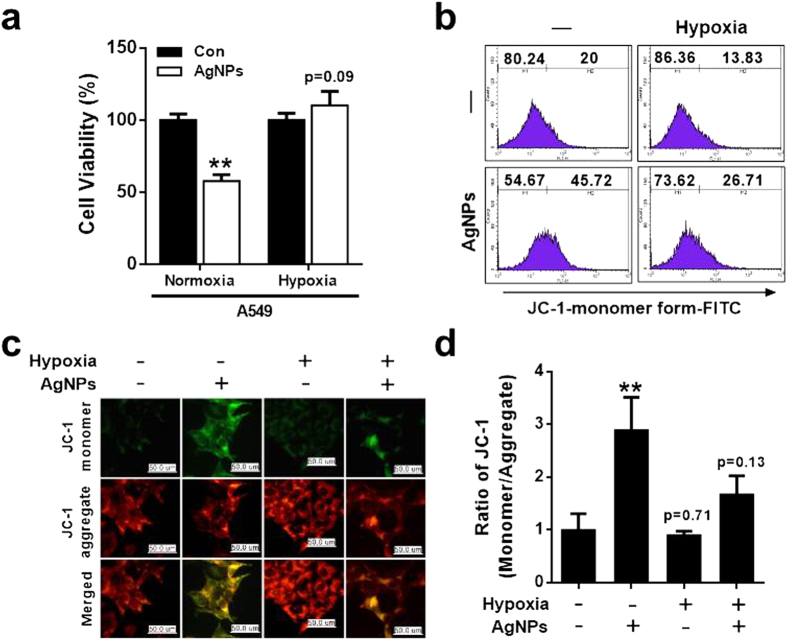
Hypoxia inhibited mitochondrial apoptosis caused by AgNPs treatment in A549 lung epithelial cells. (**a**) Cell viability was measured using the Cell Counting Kit-8 (CCK-8). The bar graph indicates the mean ± SEM (*n* = 3). *P < 0.05, **P < 0.01, significant differences between control and each treatment group. (**b**) Cells were exposed to 32.33 μg/mL of AgNPs (24 h) with or without 12 h hypoxia pre-exposure. JC-1 monomer formation (green) was measured using flow cytometry. M2 represents the population of JC-1 monomeric cells. (**c**) Representative images of JC-1-aggregate formation in cells treated as described in (**b**). Treated cells were measured for JC-1 aggregate (red) and monomer (green) formation by confocal microscopy analysis. Scale bar = 50 μm. (**d**) The bar graph indicates the JC-1-monomer/JC-1-aggregate formation ratio. **P < 0.01, significant differences between control and each treatment group.

We next assessed whether hypoxia had an inhibitory effect on AgNPs-induced oxidative stress and mitochondria-mediated apoptosis by using JC-1. JC-1 is a lipophilic, cationic dye that can selectively enter the mitochondria and reversibly change color from green to red as membrane potential increases^39^. In healthy cells with high mitochondrial ΔΨm, JC-1 spontaneously forms complexes known as J-aggregates that show intense red fluorescence. In contrast, in apoptotic or unhealthy cells with low ΔΨm, JC-1 remains in the monomeric form, showing only green fluorescence. A549 cells were incubated with AgNPs at an IC_50_ concentration of 32.33 μg/mL with or without pre-exposure to hypoxia for 12 h (Fig. 2a). The results showed that exposure to hypoxia prevented AgNPs-induced cell death.

To investigate the effect of hypoxia on AgNPs-induced mitochondrial damage, mitochondrial transmembrane potential (MTP) was evaluated in cells exposed to AgNPs with or without hypoxia treatment and labeled with the fluorescent dye JC-1. After AgNPs exposure, the number of JC-1 monomer-positive cells increased, indicating that MTP decreased. Pre-exposure of AgNPs-treated cells to hypoxia decreased the population of JC-1 monomer-positive cells, indicating that MTP levels were normal or high (Fig. 2b). These results are confirmed by fluorescence microscopy analysis (Fig. 2c). Consistently, treatment with AgNPs resulted in a high JC-1 monomer/aggregate ratio, indicating mitochondrial damage and exposure to hypoxia decreased AgNPs-mediated mitochondrial damage (Fig. 2d). These results suggest that hypoxia exposure inhibits AgNPs-induced, mitochondria-mediated apoptosis.

### Effect of hypoxia on AgNPs-induced ROS generation

**Figure 3:**
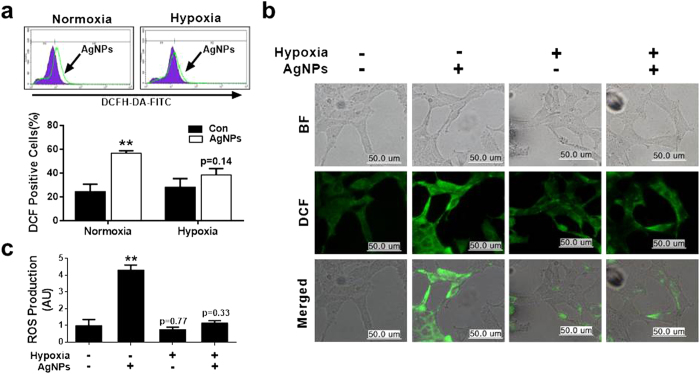
Hypoxia decreased AgNPs-mediated oxidative stress. (**a**) AgNPs-treated cells were measured for DCFH-DA-FITC by flow cytometry. M2 represents the population of DCFH-DA-FITC-positive cells. The bar graph indicates the mean ± SEM (*n* = 3). (**b**) Representative images of DCFH-DA-FITC in cells treated as described in (**a**). Treated cells were measured for DCFH-DA-FITC by fluorescence microscopy analysis. Scale bar = 50 μm. (**c**) Production of ROS measured by flow cytometry as described in (**a**). **P < 0.01 compared with the control.

Next, we investigated the relationship between increased mitochondrial damage induced by AgNPs and oxidative stress. ROS production was evaluated in A549 cells exposed to AgNPs with or without hypoxia pretreatment. After AgNPs treatment, 60% of the cells were DCF-positive, indicating high ROS production. Hypoxia pre-exposure reduced the percentage of DCF-positive cells, indicating that ROS generation was reduced (Fig. 3a). These results were confirmed using fluorescence microscopy methods (Fig. 3b). Consistently, ROS generation induced by AgNPs resulted in high FITC fluorescence intensity, indicating an increased susceptibility to oxidative stress, whereas pre-exposure to hypoxia blocked AgNPs-induced oxidative stress (Fig. 3c). Collectively, these data suggest that hypoxia inhibits mitochondria-induced apoptosis caused by AgNPs-induced oxidative stress.

### Role of HIF-1α in AgNPs-induced, mitochondria-mediated apoptosis

**Figure 4:**
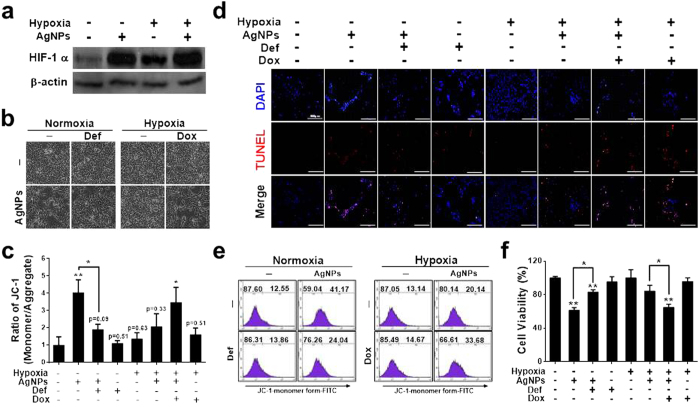
Hypoxia attenuated AgNPs-mediated mitochondrial apoptosis via regulation of HIF-1α. (**a**) AgNPs**-**treated cells were assessed for HIF-1α production by western blot analysis. Results were normalized to β-actin. (**b**) A549 cells were incubated with AgNPs with or without DEF and DOX under various oxygen conditions. Treated cells were photographed under a light microscope (200×). (**c**) Cell viability was measured using the CCK-8 assay. Viability of control cells was set to 100%, and viability relative to the control is shown. The bar graph indicates the mean ± SEM (*n* = *3*). *P < 0.05, significant differences between control and each treatment group. (**c**) Representative images of Tunel assay in cells treated as described in (**c**). Tunel analyses were carried out in triplicate for each sample. The treated cells were photographed under a fluorescence microscope (200×). (**e**) FACS analysis of MTP conversion in A549 cells treated as described in (**b**). The treated cells were measured in the JC-1 monomeric form (green) by flow cytometry. Cells were treated with JC-1; for normal MTP and low MTP in the monomeric form in the cytoplasm, JC-1 shows green fluorescence. M2 represents the population of JC-1 monomeric cells. (**f**) The bar graph indicates the J-719 monomer/J-aggregate formation ratio. *P < 0.05, **P < 0.01, significant differences between 720 control and each treatment group.

Hypoxia plays an anti-apoptotic role by regulating the expression of anti- and pro-apoptotic proteins in HIF-1α-dependent and HIF-1α-independent manners. To understand the effects of hypoxia on the sensitivity of cells to AgNPs-induced apoptosis, the expression of HIF-1α was measured by western blot analysis. Cells were cultured in the presence or absence of AgNPs under normoxic or hypoxic conditions. The results showed that HIF-1α expression was upregulated after AgNPs treatment under both hypoxic and normoxic conditions (Fig. 4a). However, HIF-1α expression was also upregulated under hypoxic conditions in the absence of AgNPs treatment (Fig. 4a). To determine whether HIF-1α plays a role in AgNPs-induced cell death, we treated the cells with an HIF-1α activator, deferoxamine (DEF), or a HIF-1α inhibitor, doxorubicin (DOX), under hypoxic/normoxic conditions. In cells treated with a combination of DEF and AgNPs, cell viability increased (Fig. 4b,c). A TUNEL assay showed that apoptosis was inhibited under normoxic conditions (Fig. 4d). In contrast, cells treated with a combination of DOX and AgNPs were strongly sensitized to AgNP treatment under hypoxic conditions (Fig. 4b–d). To determine the correlation between HIF-1α activity and the mitochondrial damage induced by AgNPs, the JC-1 assay was performed. As shown in Fig. 4e, the JC-1 monomer population increased when the cells were treated with AgNPs, but treatment with the hypoxia-mimicking compound DEF attenuated the AgNPs-induced increase in the JC-1 monomer population. Under hypoxic conditions, combined treatment with DOX and AgNPs increased the JC-1 monomer populations. These results were confirmed by fluorometric analyses (Fig. 4f). These results indicate that hypoxia inhibits AgNPs- and DOX-induced mitochondrial damage.

### HIF-1α inhibited AgNPs-induced, mitochondria-mediated apoptosis by regulating autophagic flux

**Figure 5:**
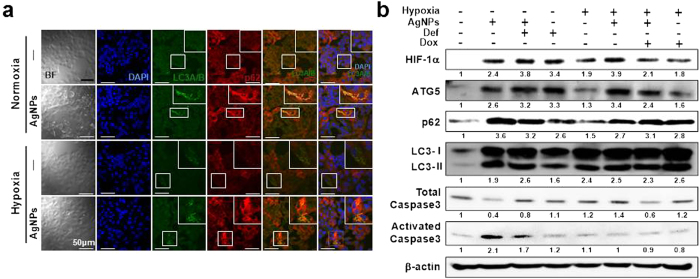
HIF-1α restored inhibition of autophagic flux caused by AgNPs treatment. (**a**) Representative images of immunocytochemistry in A549 cells treated as described in Materials and Methods. The treated cells were immunostained with DAPI (blue), LC3 (green), and p62 antibody (red). Fluorescence microscopy analyses were carried out in triplicate for each sample. (**b**) Western blot analysis of HIF-1α, ATG5, p62, LC3, and caspase-3 from A549 cells treated with 32.33 μg/mL of AgNPs, 2.5 μM DEF, or 10 nM DOX under hypoxic and normoxic conditions. β-actin was used as a loading control.

Since HIF-1α regulates autophagy activation, we measured the expression of ATG5, LC3-II, and p62 in AgNP-treated or untreated cells under hypoxic and normoxic conditions to determine the relationship between HIF-1α and autophagy-related proteins. As shown in Fig. 5a, immunocytochemistry results revealed that treatment of AgNPs increased LC3 and p62 fluorescence, indicating inhibition of autophagic flux; this inhibitory effect of AgNPs treatment was blocked by hypoxia exposure. Similarly, an immunoblot assay showed that AgNPs treatment increased the expression of ATG5, LC3-II, p62, and activated caspase-3 relative to expression in the control group under normoxic conditions. However, in AgNPs-treated cells, exposure to hypoxia decreased the expression of activated caspase-3 and p62 and increased the expression of ATG5 and LC3-II (Fig. 5b). Additionally, the hypoxia-inducer DEF decreased AgNPs-induced p62 and activated caspase-3 protein expressions and increased ATG5 and LC3-II protein expression under normoxic conditions. However, under hypoxia, DOX treatment decreased ATG5 protein expression and increased p62 and activated caspase-3 protein expressions in the presence of AgNPs (Fig. 5b). These data indicate that HIF-1α alleviates the inhibition of autophagy activation induced by AgNPs-mediated cell damage.

### Effect of HIF-1α silencing on the inhibitory effects of hypoxia on AgNPs-induced apoptosis

**Figure 6:**
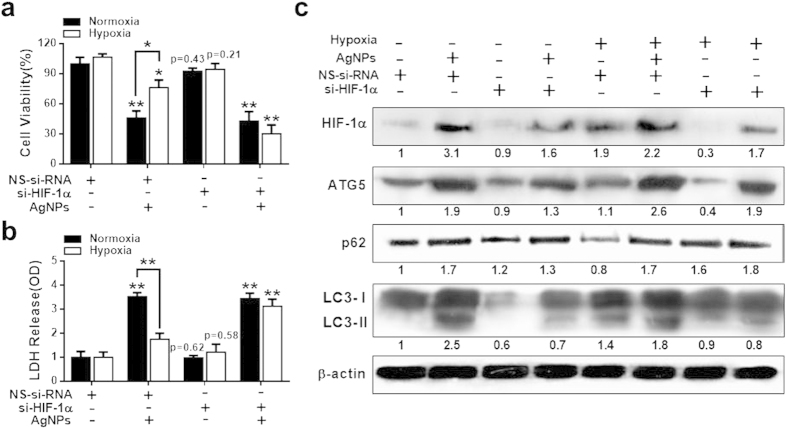
HIF-1α knockdown disrupted hypoxia-mediated autophagic flux and anti-apoptosis against AgNPs. (**a**) HIF-1α siRNA or NSsiRNA (nonspecific siRNA)-transfected A549 cells were incubated with 32.33 μg/mL AgNPs for 24 h after exposure to hypoxia for 12 h. Cell viability was measured using the CCK-8 assay. Viability of control cells was set to 100%, and viability relative to the control is presented. The bar graph indicates the mean ± SEM (*n* = *3*). *P < 0.05, significant differences between control and each treatment group. (**b**) The bar graph indicates the measured release of LDH into the cell culture supernatant from damaged cells. **P < 0.01 significant differences between control and each treatment group. (**c**) Treated cells were assessed for HIF-1α, ATG5, p62, and LC3 production by western blot analysis. Results were normalized to β-actin.

We sought to downregulate autophagy by targeting the essential autophagy gene HIF-1α. Cells were transfected with HIF-1α siRNA or control siRNA and then incubated under normoxic or hypoxic conditions in the presence or absence of AgNPs. We used siRNA to decrease the levels of HIF-1α in cells and found that HIF-1α protein levels were considerably depleted. Cell viability, lactate dehydrogenase (LDH) leakage, and the expression of HIF-1α, ATG5, LC3-II, and p62 were assessed. AgNPs treatment decreased cell viability and increased LDH leakage in HIF-1α siRNA-transfected cells compared to in the negative control siRNA group under hypoxic conditions (Fig. 6a,b). When the cells were treated with AgNPs under hypoxic conditions, cell viability was lower compared to untreated and the level of LDH leakage was higher in HIF-1α siRNA-transfected cells than in control siRNA-transfected cells (Fig. 6a,b). These results demonstrate that HIF-1α play an important role in AgNPs-induced apoptosis under hypoxic conditions.

Western blotting results showed that ATG5, LC3-II, and p62 protein expression increased when A549 cells were exposed to AgNPs. However, after HIF-1α gene knockdown, only LC3-II expression showed a considerable change under normoxic conditions (Fig. 6c). Interestingly, under hypoxic conditions, AgNPs treatment increased ATG5, p62, and LC3-II expression in control siRNA-treated cells, but did not affect p62 expression in HIF-1α knockdown cells (Fig. 6c). However, HIF-1α knockdown reduced the AgNPs-induced upregulation of ATG5 and LC3-II expression. These results are consistent with the hypothesis that regulation of HIF-1α modulates AgNPs-induced apoptosis by affecting autophagy activation.

### Role of LC3-II, ATG5, and p62 in AgNP-induced apoptosis

**Figure 7:**
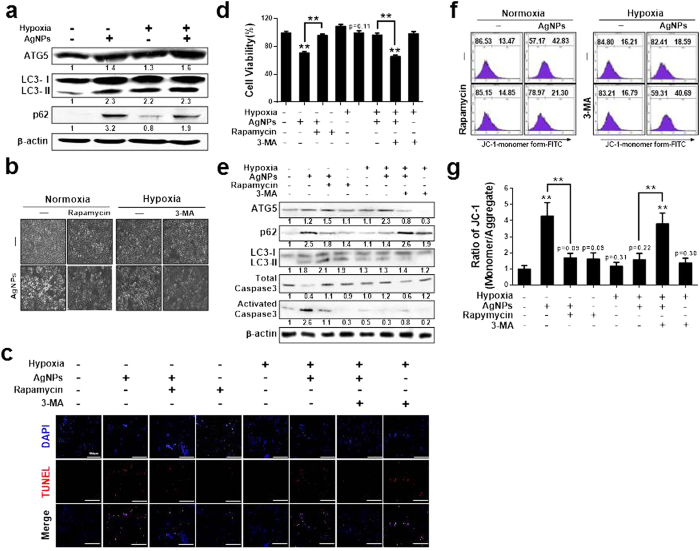
Hypoxia-induced autophagic flux attenuated AgNPs-mediated mitochondrial apoptosis. (**a**) AgNPs-treated cells were assessed for ATG5, LC3, and p62 production by western blot analysis. Results were normalized to β-actin. (**b**) A549 cells were incubated with AgNPs with or without rapamycin and 3-MA under various oxygen conditions. The treated cells were photographed under a light microscope (200×). (**c**) Representative images of fluorescence microscopy analyses in cells treated as described in (**d**). Fluorescence microscopy analyses were carried out in triplicate for each sample. (**d**) Cell viability was measured using the CCK-8 assay. Viability of control cells was set to 100%, and viability relative to the control is presented. (**g**) The bar graph indicates the J-monomer/J-aggregate formation ratio. *P < 0.05, **P < 0.01, significant differences between control and each treatment group. (**e**) Western blot analysis of HIF-1α, ATG5, p62, and LC3 from A549 cells treated as described in (**b**), β-actin was used as a loading control. (**f**) FACS analysis of MTP conversion in A549 cells treated as described in (**b**). Cells were treated with JC-1; for normal MTP and low MTP, JC-1 in the monomeric form in the cytoplasm shows green fluorescence. M2 represents the population of JC-1 monomeric cells. **g)** The bar graph indicates the J-monomer/J-aggregate formation ratio. *P < 0.05, **P < 0.01, significant differences between control and each treatment group.

To understand the mechanistic role of autophagy induction in the response to hypoxia and AgNPs treatment, we used rapamycin (Rap) and 3-methyladenine (3-MA) to activate and inhibit autophagy activation, respectively, under hypoxic/normoxic conditions. AgNPs treatment increased ATG5, LC3-II, and p62 protein levels, indicate inhibition of autophagic flux, relative to the levels in control cells under normoxic conditions. However, hypoxia exposure attenuated the AgNPs-induced increase in p62 protein expression; indicate activation of autophagic flux (Fig. 7a). In cells pre-exposed to Rap and then treated with AgNPs, apoptosis was inhibited under normoxic conditions. In contrast, cells pre-exposed to 3-MA and then treated with AgNPs were strongly sensitized to AgNPs treatment under hypoxic conditions (Fig. 7b–d).

Treatment with the autophagy inducer Rap dose-dependently increased LC3-II and decreased p62 expression in cells (Supplementary Fig. 2). Additionally, the autophagy inhibitor 3-MA increased activated caspase-3 and p62 expression and decreased LC3-II expression in cells treated with AgNPs under hypoxic conditions. In contrast, Rap pretreatment increased ATG5 and LC3-II protein levels and decreased p62 and activated caspase-3 protein levels in cells treated with AgNPs under normoxic conditions, indicating that activating autophagic flux prevents AgNPs-mediated apoptosis under these conditions (Fig. 7e). Additionally, the JC-1 assay showed that autophagic flux affected AgNPs-mediated mitochondrial damage (Fig. 7f,g). The JC-1 monomer population increased when the cells were treated with AgNPs, but treatment with the autophagy inducer Rap attenuated the AgNPs-induced increase in the JC-1 monomer population. In addition, 3-MA treatment increased the JC-1 monomer population under hypoxic conditions. These results were confirmed by measuring the MTP values by using fluorometric methods (Fig. 7g).

### ATG5 knockdown promotes AgNPs-induced apoptosis

**Figure 8:**
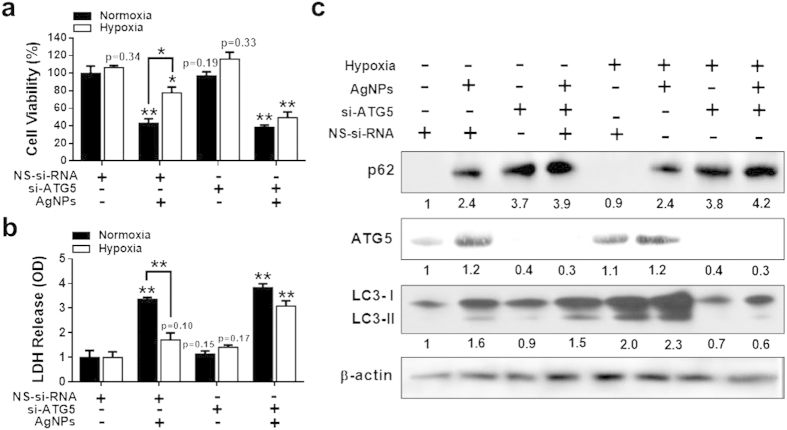
ATG5 knockdown blocked hypoxia-induced autophagic activation and anti-apoptotic effect against AgNPs treatment. (**a)** Cell viability was measured using the CCK-8 assay. Viability of control cells was set to 100%, and viability relative to the control is presented. (**b**) Bar graph indicated that the release of LDH into the cell culture supernatant from damaged cells was measured. (**c**) The treated cells were assessed for ATG5, p62, and LC3 production by western blot analysis. Results were normalized to β-actin. The bar graph indicates the mean ± SEM (*n* = *3*). *P < 0.05, significant differences between control and each treatment group.

To determine whether ATG5 suppresses AgNPs-mediated mitochondrial damage by activating autophagic flux, an ATG5 RNA interference oligomer (ATG5 siRNA) was used to knock down ATG5 expression in cells; this intervention reduced ATG5 protein levels considerably. Treatment with ATG5 siRNA attenuated the suppressive effect of hypoxia exposure on AgNPs-induced apoptosis, as determined from the reduced cell viability and increased LDH leakage observed in ATG5 siRNA-treated cells versus negative control siRNA-treated cells (Fig. 8a,b; Supplementary Fig. 3).

Western blot analysis showed that ATG5 and p62 protein levels increased in AgNPs-treated cells exposed to hypoxia. However, ATG5 knockdown increased p62 expression and decreased ATG5 and LC3-II expression (Fig. 8c). Collectively, these results showed that loss of ATG5 decreased autophagic flux and that loss of ATG5 in combination with AgNPs further reduced cell viability under hypoxic conditions. ATG5 knockdown enhanced the sensitivity of cells to AgNPs to a similar extent under normoxic and hypoxic conditions. The results indicate that autophagy can be targeted to increase the effectiveness of cancer treatment.

## Discussion

Most human solid tumors contain a substantial fraction of cells that are hypoxic, and tumor hypoxia is often associated with resistance to chemotherapy, immunotherapy, and radiotherapy^40^,^41^,^42^. Previous studies have suggested that cellular responses to hypoxia depend not only on the severity and duration of decreased oxygen availability but also on the cell type^43^,^44^. Several studies have shown that HIF-1α can induce autophagy under hypoxic conditions. Autophagy is increasingly recognized as a contributor to the malignant phenotype and as a possible mechanism for treatment failure in cancer^45^,^46^. Therefore, we investigated the roles of autophagy and hypoxia in AgNPs-induced apoptosis in A549 lung epithelial cancer cells.

Several research groups have described the toxic effects of AgNPs in various cell types, including prokaryotic and eukaryotic cells, and the potential use of AgNPs in oncologic therapy^10^. Cancer cells are more sensitive to AgNPs-induced toxicity than are non-cancer cells^47^,^48^. Moreover, Sriram *et al*.^11^ demonstrated the efficacy of biologically synthesized AgNPs as antitumor agents by using Dalton’s lymphoma ascites cell lines *in vitro* and *in vivo*. According to previous studies, the pH of tumor cells are slightly acidic compared to normal cells, and the release of silver ions from AgNPs is pH-dependent (lower pH, higher release of ions). To support information, Mukherjee *et al*. reported that an acidic tumor environment facilitated the release of anticancer phytoconstituents from biosynthesized silver nanoconjugates to help further increase the anticancer activity of biologically synthesized AgNPs^49^.

Hypoxia occurs during acute and chronic vascular diseases, pulmonary diseases, and cancer, leading to apoptotic or necrotic cell death^50^. Although hypoxia can directly promote malignant progression, HIF-1α may interfere with cytotoxic tumor therapies by regulating autophagy under hypoxic conditions. The exact mechanisms by which hypoxia promotes the resistance to apoptosis in cancer cells are not well understood. Therefore, in this study, we tested whether hypoxia reduces the AgNPs-induced loss of MTP and ROS production. Our results showed that AgNPs treatment markedly decreased MTP and increased ROS production in A549 cells under normoxic conditions. However, hypoxia reduced the AgNPs-induced depolarization of MTP, which agrees with the results of a previous study demonstrating that hypoxia reduced the TRAIL-induced increase in the JC-1 monomer population in A549 cells^51^. In addition, our results showed that hypoxia reduced AgNPs-induced ROS generation, likely via the decreased oxygen supply, suggesting that oxygen is an important substrate for AgNPs-induced ROS formation, particularly in lung cell lines exposed to high levels of oxygen. This hypothesis is supported by Kim *et al*.^52^, whose results suggested that nonlethal continuous hypoxia protects lung epithelial cells against paraquat-induced toxicity by reducing the oxygen supply and thereby reducing the generation of free radicals that contribute to cell death.

Because HIF-1α is an important regulator of the adaptive response to hypoxia^53^, we investigated its role in hypoxia-induced resistance to AgNPs-mediated apoptosis. Western blot data indicated that AgNPs induced HIF-1α expression under normoxic and hypoxic conditions. However, DEF pretreatment attenuated the AgNPs-induced decrease in cell viability, MTP, and apoptotic cell morphology, suggesting that DEF inhibits AgNPs-induced cell death. In contrast, the combination of DOX and AgNPs severely compromised the protective effects of hypoxia. Similarly, HIF-1α knockdown in A549 cells also reduced the effect of hypoxia on AgNPs-mediated cytotoxicity. These results provide strong evidence for the role of HIF-1α-induced autophagy in hypoxia-induced chemoresistance to several chemotherapeutic agents in cancer cell lines.

Both hypoxia and nutrient deprivation induce autophagy, which has recently emerged as a critical cellular process in cancer cell survival^26^,^46^. The ATG5 cleavage product, but not intact ATG5, which participates in autophagy, can significantly affect apoptosis^54^. LC3 is a mammalian homolog of the yeast Atg8 protein. During autophagy, the cytoplasmic form (LC3 I) is processed and recruited to autophagosomes, where LC3-II is generated by site-specific proteolysis and lipidation near the C-terminus^55^. Among autophagy-related proteins, p62 is degraded by autophagy. p62 may link ubiquitinated proteins to the autophagic machinery to enable their degradation in the lysosome. Since decreased levels of p62 protein are observed during autophagy induction, p62 can be used as a marker to examine autophagic flux^56^. ATG5 may be important in both apoptosis and autophagy. Our results showed that hypoxia increased the expression of ATG5 and LC3-II and decreased the expression of p62 in AgNPs-treated cells (Fig. 7a). Studies with an HIF-1α activator (DEF) and inhibitor (DOX) and with HIF-1α siRNA clearly showed that hypoxia conferred resistance to AgNPs-mediated cytotoxicity via HIF-1α-induced autophagy. Our data also showed that Rap, an autophagy activator, severely compromised the ability of AgNPs to decrease cell viability and MTP and upregulate p62, while autophagy inhibition increased the anticancer efficacy of AgNPs. The inhibitory effect of autophagy induction on anticancer therapy is supported by the findings of Gupta *et al*.^57^ and Hollomon *et al*.^58^, who observed the opposite effect of ATG5 knockdown-mediated autophagy inhibition on camptothecin-induced cytotoxicity in osteosarcoma cells. However, our results showed that ATG5 knockdown increased the sensitivity to AgNPs in A549 cells.

The association between autophagy and AgNPs-regulated or AgNPs-nonregulated cell death has been unclear. Based on our experimental results, autophagy and apoptosis induced by AgNPs may be coincident or an antagonistic effect, depending on the experimental conditions, or may share cross-talk between signal transduction elements involved in autophagy. Autophagy may protect against toxicities such as endogenous physiological stress or exogenous stimuli induced by AgNPs.

In conclusion, biologically derived AgNPs showed significant inhibitory effects in A549 cells. Further, the inhibitory effect of AgNPs on cell viability, membrane leakage, membrane potential, and ROS generation was attenuated by hypoxia-mediated autophagy activation, which reduced the cytotoxic efficacy of AgNPs in A549 cells in an HIF-1α-dependent manner and through the regulation of ATG5, LC3-II, and p62. Despite emerging evidence for a role of AgNPs in apoptosis, the biochemical and signaling mechanisms by which hypoxia inhibits AgNPs through the activation of HIF-1α are not fully understood. The results of our study suggest that hypoxia conditions can alleviate AgNPs-induced apoptosis in cancer cells via the HIF-1α-mediated autophagy pathway (Supplementary Fig. 4), which is an important regulatory event in tumor cells. These findings indicate that autophagy can regulate AgNPs-induced cell death. Thus, strategies targeting HIF-1α may be effective in cancer therapy.

## Materials and Methods

### Synthesis and characterization of AgNPs

AgNPs were synthesized according to Gurunathan *et al*.^59^ Briefly, *B. flexus* was grown in a 500-mL Erlenmeyer flask containing nutrient broth (NB) medium. The flask was incubated for 21 h in a shaker set at 120 rpm and 37 °C. After the incubation period, the culture was centrifuged at 10,000 rpm, and the supernatant was used for AgNP synthesis. The culture supernatant was incubated with AgNO_3_ solution at a concentration of 5 mM for 6 h. The extracellular synthesis of AgNPs was monitored by visual inspection of the test tubes for a change in the color of the culture medium from clear, light yellow to brown. AgNPs were characterized according to previously described methods^59^. The synthesized AgNPs were dissolved in double-distilled water and stored at room temperature.

### Cell culture and exposure to hypoxic conditions

Human alveolar basal epithelial cells (A549) and human epithelial lung cells (L132) were obtained from the Korean Cell Bank (Seoul, Korea) and cultured in RPMI 1640 medium supplemented with 10% fetal bovine serum (FBS) and 1 mL of penicillin-streptomycin solution were added to 100 mL of cell culture media for a final concentration of 50–100 IU/mL penicillin and 50–100 μg/mL streptomycin at 5% CO_2_ and 37 °C. At 75% confluence, the cells were harvested using 0.25% trypsin and were subcultured in 75-cm^2^ flasks, 6-well plates, or 96-well plates depending on the experiment. The cells were allowed to adhere for 24 h before treatment. The medium was replaced three times per week, and the cells were passaged at subconfluency.

### Hypoxia exposure and treatments

The hypoxic conditions and treatments described previously^60^ were followed with appropriate modifications. Briefly, hypoxia was induced by placing logarithmic phase, subconfluent monolayer cultures in a hypoxia incubator chamber and equilibrated for 30 min. The hypoxia incubator was used to create a low oxygen environment; a gas mixture of 5% O_2_, 5% CO_2_, and 90% N_2_ flowed into the hypoxia incubator. Ambient air was evacuated through an outlet tube, and O_2_ flowed through the chamber for 2–3 min to maintain the desired O_2_ tension. The cultures were maintained under hypoxic conditions for 24 h. Control cells were grown in normal oxygen for the same duration. After incubation, the medium and cells were collected within 2–3 min to avoid re-oxygenation of the cells. Cells were pretreated in triplicate in 6-well plate wells at 37 °C for 24 h with or without AgNPs or other reagents. Cells were treated with 5 μM of deferoxamine, 10 μM of doxorubicin, and 100 nM of rapamycin and 1 mM of 3-methyladenine, respectively.

### Cell viability and cytotoxicity assays

Cell viability was measured using Cell Counting Kit-8 (CCK-8; CK04-01, Dojindo Laboratories, Kumamoto, Japan). Briefly, A549 and L132 cells were plated onto 96-well flat-bottom culture plates containing various concentrations of AgNPs. All cultures were incubated for 24 h at 37 °C (5% CO_2_ in a humidified incubator). CCK-8 solution (10 μL) was added to each well, and the plate was incubated for another 2 h at 37 °C. The absorbance was measured at 450 nm by using a microplate reader (Multiskan FC; Thermo Fisher Scientific Inc., Waltham, MA, USA). Cytotoxicity was assessed using supernatants from the medium in LDH assays. An LDH Cytotoxicity Detection kit was used according to the manufacturer’s protocol, and the absorbance was measured at 490 nm using a microplate reader. Concentrations of AgNPs showing a 50% reduction in cell viability (i.e., half-maximal inhibitory concentration [IC_50_] values) were calculated and then used for further experiments.

### DCFH-DA assay

A549 cells were incubated in RPMI-1640 medium containing 10 μM 2′,7′-dichlorodihydrofluorescein diacetate (DCFH-DA; D6883, Sigma-Aldrich, St. Louis, MO, USA) at 37 °C for 30 min. The cells were washed with phosphate-buffered saline (PBS), lysed in lysis buffer (25 mM HEPES; pH 7.4, 100 mM NaCl, 1 mM EDTA, 5 mM MgCl_2_, 0.1 mM dithiothreitol, and a protease inhibitor mixture), and transferred to a clear 96-well plate. Fluorescence was measured with excitation and emission wavelengths of 488 and 515 nm, respectively, by using a SpectraMAX Gemini EM microplate reader (Molecular Devices, Sunnyvale, CA, USA). DCFH-DA-positive cell populations were identified using a FACSCalibur cell analyzer (BD Biosciences, Franklin Lakes, NJ, USA). The cells were also cultured on cover slips in RPMI medium containing 10 μM DCFH-DA for 30 min at 37 °C. The cells were then washed with PBS, mounted using VECTASHIELD fluorescent medium, and visualized by fluorescence microscopy.

### JC-1 assay

The change in MTP was evaluated using the cationic fluorescent indicator JC-1 (T-3168; Molecular Probes, Eugene, OR, USA). J-aggregates in intact mitochondria fluoresce red with emission at 583 nm, and J-monomers in the cytoplasm fluoresce green with emission at 525 nm and an excitation wavelength of 488 nm. A549 cells were incubated in RPMI medium containing 10 μM JC-1 at 37 °C for 15 min, washed with PBS, and transferred to a clear 96-well plate. The fluorescence intensity was measured. Cell populations containing the JC-1 monomer were identified using a FACSCalibur cell analyzer. Cells were also cultured on cover slips, incubated in DMEM containing 10 μM JC-1 at 37 °C for 15 min, and then washed with PBS. Finally, the cells were mounted using VECTASHIELD fluorescent medium and visualized by fluorescence microscopy.

### Flow cytometry analysis

A549 cells were trypsinized and aliquoted. Aliquots containing 2.5–5.0 × 10^5^ cells were stained for 30 min with the fluorescent dyes JC-1 and DCFH-DA. Thereafter, the cells were washed in PBS, centrifuged at 1200 × *g*, and resuspended in maintaining buffer (PBS containing 1% FBS). Cells were characterized using a FACSCalibur cell analyzer, and the data were analyzed using CellQuest software (BD Biosciences). For each run, 10^4^ events were collected. We counted at least 10,000 cells/tube for flow cytometry analysis in each experiment, respectively.

### RNA interference

A549 cells were transfected with ATG5 small interfering RNA (siRNA; oligo ID s18159; 4392420, Ambion/Life Technologies, Carlsbad, CA, USA) or HIF-1α siRNA (oligo ID s6541; 4390824, Ambion/Life Technologies) using Lipofectamine 2000 (11668019, Life Technologies) according to the manufacturer’s instructions. After a 48-h culture, knockdown efficiency was assessed at the protein level by immunoblot analysis. Nonspecific siRNA (Negative Control #1 siRNA; AM4636, Ambion/Life Technologies) was used as a negative control.

A549 cells were lysed in buffer containing 25 mM HEPES, pH 7.4, 100 mM NaCl, 1 mM EDTA, 5 mM MgCl_2_, 0.1 mM dithiothreitol, and a protease inhibitor mixture. Equal amounts of lysate were resolved by 10–15% sodium dodecyl sulfate-polyacrylamide gel electrophoresis (SDS-PAGE) and electrophoretically transferred to a nitrocellulose membrane. Immunoreactivity was detected by sequential incubations with horseradish peroxidase-conjugated secondary antibodies and enhanced chemiluminescence reagents.

### Immunocytochemistry

Cells were cultured on glass cover slips, washed with PBS, and fixed in cold acetone for 90 s at room temperature. The cells were washed again with PBS, blocked with 5% FBS in Tris-buffered saline containing Tween 20 (TBST), and incubated with 2 μg/mL anti-mouse-p-62 monoclonal antibody and 2 μg/mL anti-rabbit-LC3 polyclonal antibody for 48 h at room temperature. Unbound antibody was removed by washing with PBS and the cells were incubated with 4 μg/mL Alexa Fluor 488 anti-rabbit FITC (for anti-LC3 antibody) and Alexa Fluor 546 anti-mouse (for anti-p62 antibody) IgG antibodies for 2 h at room temperature. Finally, the cells were mounted using the VECTASHIELD fluorescent medium visualized by fluorescence microscopy. Fluorescence microscopy analyses were carried out in triplicate for each sample. We counted at least 100 cells/well for confocal fluorescence microscopy in each experiment.

### Statistical analysis

All data were expressed as the mean ± standard error of the mean (SEM) and compared using one-way analysis of variance and Turkey’s test as the *post-hoc* comparison test (*P < 0.05, **P < 0.01) by using GraphPad Prism software, version 5.0 (La Jolla, CA, USA).

## Additional Information

**How to cite this article**: Jeong, J.-K. *et al*. Hypoxia-mediated autophagic flux inhibits silver nanoparticle-triggered apoptosis in human lung cancer cells. *Sci. Rep.* **6**, 21688; doi: 10.1038/srep21688 (2016).

## Supplementary Material

Supplementary Information

## References

[b1] Chen X. & Schluesener H. J. Nanosilver: a nanoproduct in medical application. Toxicol Lett 176, 1–12 (2008). 18022772 10.1016/j.toxlet.2007.10.004

[b2] Yamashita K. . Silica and titanium dioxide nanoparticles cause pregnancy complications in mice. Nat Nanotechnol 6, 321–328 (2011). 21460826 10.1038/nnano.2011.41

[b3] Asharani P. V., Hande M. P. & Valiyaveettil S. Anti-proliferative activity of silver nanoparticles. BMC Cell Biol 10, 65 (2009). 19761582 10.1186/1471-2121-10-65 PMC2759918

[b4] Kim S. & Ryu D. Y. Silver nanoparticle-induced oxidative stress, genotoxicity and apoptosis in cultured cells and animal tissues. J Appl Toxicol 33, 78–89 (2013). 22936301 10.1002/jat.2792

[b5] Schrand A. M., Braydich-Stolle L. K., Schlager J. J., Dai L. & Hussain S. M. Can silver nanoparticles be useful as potential biological labels? Nanotechnology 19, 235104 (2008). 21825779 10.1088/0957-4484/19/23/235104

[b6] Lee Y. H. . Cytotoxicity, oxidative stress, apoptosis and the autophagic effects of silver nanoparticles in mouse embryonic fibroblasts. Biomaterials 35, 4706–4715 (2014). 24630838 10.1016/j.biomaterials.2014.02.021

[b7] Tiwari D. K., Jin T. & Behari J. Dose-dependent *in-vivo* toxicity assessment of silver nanoparticle in Wistar rats. Toxicol Mech Methods 21, 13–24 (2011). 21080782 10.3109/15376516.2010.529184

[b8] Rahman M. F. . Expression of genes related to oxidative stress in the mouse brain after exposure to silver-25 nanoparticles. Toxicol Lett 187, 15–21 (2009). 19429238 10.1016/j.toxlet.2009.01.020

[b9] Herzog F. . Exposure of silver-nanoparticles and silver-ions to lung cells *in vitro* at the air-liquid interface. Particle and Fibre Toxicology 10, 11 (2013). 23557437 10.1186/1743-8977-10-11 PMC3639923

[b10] Pimentel A., Velez M., Barahona L. J., Swords R. & Lekakis L. New prospects for drug development: the hedgehog pathway revealed. Focus on hematologic malignancies. Future Oncol 9, 681–697 (2013). 23647297 10.2217/fon.13.10

[b11] Sriram M. I., Kanth S. B. M., Kalishwaralal K. & Gurunathan S. Antitumor activity of silver nanoparticles in Dalton’s lymphoma ascites tumor model. Int J Nanomed 5, 753–762 (2010). 10.2147/IJN.S11727 PMC2962271 21042421

[b12] Semenza G. L. HIF-1, O-2, and the 3 PHDs: How animal cells signal hypoxia to the nucleus. Cell 107, 1–3 (2001). 11595178 10.1016/s0092-8674(01)00518-9

[b13] Huang L. E. & Bunn H. F. Hypoxia-inducible factor and its biomedical relevance. J Biol Chem 278, 19575–19578 (2003). 12639949 10.1074/jbc.R200030200

[b14] Park S. Y., Billiar T. R. & Seol D. W. Hypoxia inhibition of apoptosis induced by tumor necrosis factor-related apoptosis-inducing ligand (TRAIL). Biochem Bioph Res Co 291, 150–153 (2002). 10.1006/bbrc.2002.6421 11829475

[b15] Haase V. H. The VHL Tumor Suppressor: Master Regulator of HIF. Curr Pharm Design 15, 3895–3903 (2009). 10.2174/138161209789649394 PMC3622710 19671042

[b16] Hu Y. . Inhibition of hypoxia-inducible factor-1 function enhances the sensitivity of multiple myeloma cells to melphalan. Mol Cancer Ther 8, 2329–2338 (2009). 19671732 10.1158/1535-7163.MCT-09-0150

[b17] Bellot G. . Hypoxia-Induced Autophagy Is Mediated through Hypoxia-Inducible Factor Induction of BNIP3 and BNIP3L via Their BH3 Domains. Molecular and Cellular Biology 29, 2570–2581 (2009). 19273585 10.1128/MCB.00166-09 PMC2682037

[b18] Iyer N. V. . Cellular and developmental control of O2 homeostasis by hypoxia-inducible factor 1 alpha. Genes & Dev 12, 149–162 (1998). 9436976 10.1101/gad.12.2.149 PMC316445

[b19] Mayes P. A. . Modulation of TRAIL-induced tumor cell apoptosis in a hypoxic environment. Cancer Biology & Therapy 4, 1068–1074 (2005). 16294025 10.4161/cbt.4.10.2255

[b20] Zhou J., Kohl R., Herr B., Frank R. & Brune B. Calpain mediates a von Hippel-Lindau protein-independent destruction of hypoxia-inducible factor-1alpha. Mol Biol Cell 17, 1549–1558 (2006). 16421254 10.1091/mbc.E05-08-0770 PMC1415322

[b21] Lee M. J., Kim J. Y., Suk K. H. & Park J. H. Identification of the hypoxia-inducible factor 1 alpha-responsive HGTD-P gene as a mediator in the mitochondrial apoptotic pathway. Mol Cell Biol 24, 3918–3927 (2004). 15082785 10.1128/MCB.24.9.3918-3927.2004 PMC387743

[b22] Singh R. . Autophagy regulates lipid metabolism. Nature 458, 1131–1135 (2009). 19339967 10.1038/nature07976 PMC2676208

[b23] Chen N. & Karantza-Wadsworth V. Role and regulation of autophagy in cancer. Biochimica et Biophysica Acta (BBA) - Mol Cell Res 1793, 1516–1523 (2009). 10.1016/j.bbamcr.2008.12.013 PMC3155287 19167434

[b24] Alirezaei M., Kemball C. C. & Whitton J. L. Autophagy, inflammation and neurodegenerative disease. European Journal of Neuroscience 33, 197–204 (2011). 21138487 10.1111/j.1460-9568.2010.07500.x PMC3059259

[b25] Hu Y. L. . Hypoxia-induced autophagy promotes tumor cell survival and adaptation to antiangiogenic treatment in glioblastoma. Cancer Res 72, 1773–1783 (2012). 22447568 10.1158/0008-5472.CAN-11-3831 PMC3319869

[b26] Esumi H. . Hypoxia and nitric oxide treatment confer tolerance to glucose starvation in a 5′-AMP-activated protein kinase-dependent manner. J Biol Chem 277, 32791–32798 (2002). 12091379 10.1074/jbc.M112270200

[b27] Papandreou I., Lim A. L., Laderoute K. & Denko N. C. Hypoxia signals autophagy in tumor cells via AMPK activity, independent of HIF-1, BNIP3, and BNIP3L. Cell Death Differ 15, 1572–1581 (2008). 18551130 10.1038/cdd.2008.84

[b28] Tang F. S. . A life-span extending form of autophagy employs the vacuole-vacuole fusion machinery. Autophagy 4, 874–886 (2008). 18690010 10.4161/auto.6556

[b29] Slobodkin M. R. & Elazar Z. The Atg8 family: multifunctional ubiquitin-like key regulators of autophagy. Essays in Biochemistry 55, 51–64 (2013). 24070471 10.1042/bse0550051

[b30] Svenning S. & Johansen T. Selective autophagy. Essays in biochemistry 55, 79–92 (2013). 24070473 10.1042/bse0550079

[b31] Lippai M. & Low P. The role of the selective adaptor p62 and ubiquitin-like proteins in autophagy. Biomed Res Int 2014, 832704 (2014). 25013806 10.1155/2014/832704 PMC4075091

[b32] Rogov V., Dotsch V., Johansen T. & Kirkin V. Interactions between autophagy receptors and ubiquitin-like proteins form the molecular basis for selective autophagy. Mol Cell 53, 167–178 (2014). 24462201 10.1016/j.molcel.2013.12.014

[b33] Ishii T., Warabi E., Siow R. C. & Mann G. E. Sequestosome1/p62: a regulator of redox-sensitive voltage-activated potassium channels, arterial remodeling, inflammation, and neurite outgrowth. Free Radical Biology & Medicine 65, 102–116 (2013). 23792273 10.1016/j.freeradbiomed.2013.06.019

[b34] Komatsu M., Kageyama S. & Ichimura Y. p62/SQSTM1/A170: physiology and pathology. Pharmacological Research 66, 457–462 (2012). 22841931 10.1016/j.phrs.2012.07.004

[b35] Lin X. . Interaction domains of p62: a bridge between p62 and selective autophagy. DNA and Cell Biology 32, 220–227 (2013). 23530606 10.1089/dna.2012.1915

[b36] Mizumura K., Choi A. M. & Ryter S. W. Emerging role of selective autophagy in human diseases. Frontiers in Pharmacology 5, 244 (2014). 25414669 10.3389/fphar.2014.00244 PMC4220655

[b37] Buckley K. M. . Rapamycin up-regulation of autophagy reduces infarct size and improves outcomes in both permanent MCAL, and embolic MCAO, murine models of stroke. Experimental & Translational Stroke Medicine 6, 8 (2014). 24991402 10.1186/2040-7378-6-8 PMC4079187

[b38] Noman M. Z., Janji B., Berchem G., Mami-Chouaib F. & Chouaib S. Hypoxia-induced autophagy: A new player in cancer immunotherapy? Autophagy 8, 704–706 (2012). 22441015 10.4161/auto.19572

[b39] Perelman A. . JC-1: alternative excitation wavelengths facilitate mitochondrial membrane potential cytometry. Cell Death Dis 3, e430 (2012). 23171850 10.1038/cddis.2012.171 PMC3542606

[b40] Teicher B. A. Hypoxia and drug resistance. Cancer Metastasis Reviews 13, 139–168 (1994). 7923547 10.1007/BF00689633

[b41] Brown J. M. & Giaccia A. J. The unique physiology of solid tumors: opportunities (and problems) for cancer therapy. Cancer Res 58, 1408–1416 (1998). 9537241

[b42] Hockel M. & Vaupel P. Tumor hypoxia: definitions and current clinical, biologic, and molecular aspects. Journal of the National Cancer Institute 93, 266–276 (2001). 11181773 10.1093/jnci/93.4.266

[b43] Cosse J. P. . Differential effects of hypoxia on etoposide-induced apoptosis according to the cancer cell lines. Molecular Cancer 6, 61 (2007). 17894897 10.1186/1476-4598-6-61 PMC2099441

[b44] Sermeus A. . Hypoxia induces protection against etoposide-induced apoptosis: molecular profiling of changes in gene expression and transcription factor activity. Molecular Cancer 7, 27 (2008). 18366759 10.1186/1476-4598-7-27 PMC2330149

[b45] Mathew L. K. & Simon M. C. mir-210: a sensor for hypoxic stress during tumorigenesis. Molecular Cell 35, 737–738 (2009). 19782023 10.1016/j.molcel.2009.09.008 PMC2869244

[b46] Selvakumaran M., Amaravadi R. K., Vasilevskaya I. A. & O’Dwyer P. J. Autophagy inhibition sensitizes colon cancer cells to antiangiogenic and cytotoxic therapy. Clinical Cancer Research 19, 2995–3007 (2013). 23461901 10.1158/1078-0432.CCR-12-1542

[b47] AshaRani P. V., Low Kah Mun G., Hande M. P. & Valiyaveettil S. Cytotoxicity and genotoxicity of silver nanoparticles in human cells. ACS Nano 3, 279–290 (2009). 19236062 10.1021/nn800596w

[b48] Gurunathan S., Raman J., Malek N. A., John P. A. & Vikineswary S. Green synthesis of silver nanoparticles using Ganoderma neo-japonicum Imazeki: a potential cytotoxic agent against breast cancer cells. Int J Nanomed 8, 4399–4413 (2013). 10.2147/IJN.S51881 PMC3833323 24265551

[b49] Mukherjee S. . Potential theranostics application of bio-synthesized silver nanoparticles (4-in-1 system). Theranostics 4, 316–335 (2014). 24505239 10.7150/thno.7819 PMC3915094

[b50] Harris A. L. Hypoxia - A key regulatory factor in tumour growth. Nat Rev Cancer 2, 38–47 (2002). 11902584 10.1038/nrc704

[b51] Lee Y. J., Lee J. H., Moon J. H. & Park S. Y. Overcoming Hypoxic-Resistance of Tumor Cells to TRAIL-Induced Apoptosis through Melatonin. Int J Mol Sci 15, 11941–11956 (2014). 25000265 10.3390/ijms150711941 PMC4139822

[b52] Kim H., Lee S. W., Baek K. M., Park J. S. & Min J. H. Continuous hypoxia attenuates paraquat-induced cytotoxicity in the human A549 lung carcinoma cell line. Exp Mol Med 43, 494–500 (2011). 21734449 10.3858/emm.2011.43.9.056 PMC3203239

[b53] Gordan J. D. & Simon M. C. Hypoxia-inducible factors: central regulators of the tumor phenotype. Curr Opin Genet Dev 17, 71–77 (2007). 17208433 10.1016/j.gde.2006.12.006 PMC3215290

[b54] Yousefi S. . Calpain-mediated cleavage of Atg5 switches autophagy to apoptosis. Nat Cell Biol 8, 1124–U1146 (2006). 16998475 10.1038/ncb1482

[b55] Tanida I., Ueno T. & Kominami E. LC3 and Autophagy. Methods Mol Biol 445, 77–88 (2008). 18425443 10.1007/978-1-59745-157-4_4

[b56] Bjorkoy G. . Monitoring Autophagic Degradation of P62/Sqstm1. Method Enzymol 452, 181–197 (2009). 10.1016/S0076-6879(08)03612-4 19200883

[b57] Gupta S. D., Gomes A., Debnath A., Saha A. & Gomes A. Apoptosis induction in human leukemic cells by a novel protein Bengalin, isolated from Indian black scorpion venom: Through mitochondrial pathway and inhibition of heat shock proteins. Chem-Biol Interact 183, 293–303 (2010). 19913524 10.1016/j.cbi.2009.11.006

[b58] Hollomon M. G., Gordon N., Santiago-O’Farrill J. M. & Kleinerman E. S. Knockdown of autophagy-related protein 5, ATG5, decreases oxidative stress and has an opposing effect on camptothecin-induced cytotoxicity in osteosarcoma cells. Bmc Cancer 13, 500 (2013). 24160177 10.1186/1471-2407-13-500 PMC3924338

[b59] Gurunathan S. . Antiangiogenic properties of silver nanoparticles. Biomaterials 30, 6341–6350 (2009). 19698986 10.1016/j.biomaterials.2009.08.008

[b60] Nagaraj N. S., Vigneswaran N. & Zacharias W. Hypoxia inhibits TRAIL-induced tumor cell apoptosis: Involvement of lysosomal cathepsins. Apoptosis 12, 125–139 (2007). 17136492 10.1007/s10495-006-0490-1 PMC5774619

