# Supplementary material for: Hypoxia-mediated autophagic flux inhibits silver nanoparticle-triggered apoptosis in human lung cancer cells
Source: Sci Rep. 2016 Feb 12;6:21688. doi: 10.1038/srep21688 (PMC4751501; doi:10.1038/srep21688)
Supplement: Supplementary Information [file srep21688-s1.doc]

**Hypoxia-mediated autophagic flux inhibits silver nanoparticle-triggered apoptosis in human lung cancer cells**

Jae-Kyo Jeong^1#^, Sangiliyandi Gurunathan^1#^, Min-Hee Kang^1^, Jae Woong Han^1^, Joydeep Das^1^, Yun-Jung Choi^1^, Deug-Nam Kwon^1^, Ssang-Goo Cho^1^, Chankyu Park^1^, Han Geuk Seo^1^, Hyuk Song^1^, and Jin-Hoi Kim^1^*

^1^Department of Animal Biotechnology, Konkuk University, Seoul 143-701, Republic of Korea.

^#^,Jae-Kyo Jeong and Sangiliyandi Gurunathan contributed equally to this work.

***For correspondence:** Professor. Jin-Hoi Kim, PhD (jhkim541@konkuk.ac.kr)

Department of Animal Biotechnology,

Konkuk University, Seoul 143-701, Republic of Korea

Tel: +82-2-450-3687, Fax: +82-2-544-4645

**Materials and Methods**

Materials

Penicillin-streptomycin solution (Life Technlogy/Gibco, 15140-112) trypsin- ethylenediaminetetraacetic acid (EDTA) solution (Life Technlogy/Gibco, 25200-072), RPMI 1640 medium (Life Technlogy/Gibco, 11875-093), and 1% antibiotic-antimycotic solution (Life Technlogy/Gibco, 15240-096) were obtained from Life Technologies/Gibco. Silver nitrate (Sigma-Aldrich, 209139), fetal bovine serum (FBS) (Sigma-Aldrich, F2442) and the LDH Cytotoxicity Detection kit (Takara Bio Inc., MK401) were purchased from Sigma-Aldrich. The antibodies used for immunoblotting targeted LC3 (Cell Signaling Technology, 4108S), ATG5 (Novus Biologicals, NB 110-53818), p62 (Enzo Diagnostics Inc., BML-PW9860-0100), HIF-1α (Enzo Diagnostics Inc., ALX-804-216-R100), Caspase3 (Cell Signaling Technology, #9662) and β-actin (Sigma-Aldrich, A3853) (**Supplementary Table 1**).

**Transmission electron microscopy (TEM) analysis**

After fixation of specimens in 2.5% glutaraldehyde (Ted Pella Inc., 18426) in phosphate-buffered saline (PBS; pH 7.2), samples were postfixed in 1% osmium tetroxide (Heraeus, 89.740.219), dehydrated in a graded series of ethanol baths and propylene oxide (Acros Organics, 149620025), and then embedded in epoxy resin (EMbed 812 containing nadic methyl anhydride, dodenyl succinic anhydride, and DMP-30) as described previously.[^50^](#_ENREF_50) Serial ultrathin sections were cut on an LKB Ultratome III (Leica, Wetzlar, Germany). Sections were stained with uranyl acetate (Ted Pella Inc., 19485) and lead citrate (Ted Pella Inc., 19314) and examined under a Hitachi H7600 electron microscope (Hitachi, Tokyo, Japan) at an accelerating voltage of 100 kV.

**Table S 1.** Source of Antibodies used in Western Blot

| Primary Antibodies | Host | Dilution | Source |
| --- | --- | --- | --- |
| LC3A/B, Autophagy marker Light Chain 3 | Rabbit | 1:1000 | Cell Signaling Technology, Danvers, MA, USA |
| ATG5, Autophagy protein 5 | Rabbit | 1:1000 | Novus Biologicals, Littleton, CO, USA |
| P62, a receptor for cargo destined to be degraded by autophagy | Mouse | 1:1000 | Abcam, Cambridge, MA, USA |
| HIF-1α, hypoxia-inducible factor-1-alpha | Mouse | 1:1000 | ENZO Diagnostics Inc, Farmingdale, NY, USA |
| Caspase3, apoptosis marker | Rabbit | 1:1000 | Cell Signaling Technology, Danvers, MA, USA |
| ß-actin | Mouse | 1:1000 | Sigma*-*Aldrich, St. Louis, MO, USA |
| Secondary Antibodies | Host | Dilution | Source |
| Anti rabbit IgG (HRP) | Goat | 1:1000 | Abcam, Cambridge, MA, USA |
| Anti mouse IgG (HRP) | Goat | 1:1000 | Santa Cruz Biotechnology, CA, USA |
| Anti rabbit IgG (Alexa Fluor 488) | Goat | 1:1000 | Invitrogen-Life tehnologies, Carlsbad CA, USA |
| Anti mouse IgG (Alexa Fluor 546) | Goat | 1:1000 | Invitrogen-Life tehnologies, Carlsbad CA, USA |

**
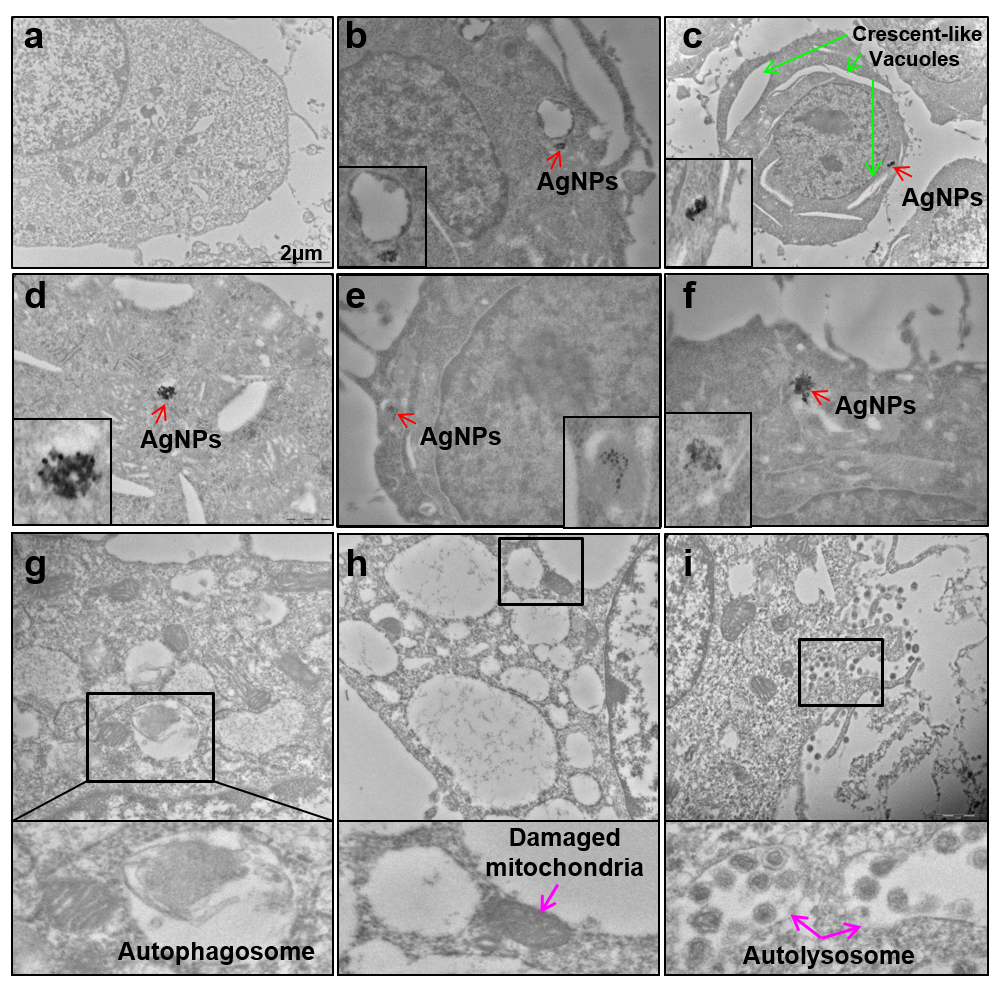
**

**Supplementary Fig. 1. Electron microscopic analysis of silver nanoparticles induced organelle damage.** A549 cells were treated with AgNPs for 24 hours. Transmission electron micrographs describing AgNPs induced autophagy in A549 cells. Cells without AgNPs **(a)**; internalization of AgNPs **(b);** formation of crescent like vacuoles **(c)**. Internalization and accumulation of AgNPs inside the cells **(d and f).** AgNPs induces formation of autophagosomes **(g)**; damaging mitochondria and formation of enlarged vacuoles **(h**); formation of autolysosomes **(i).**

**
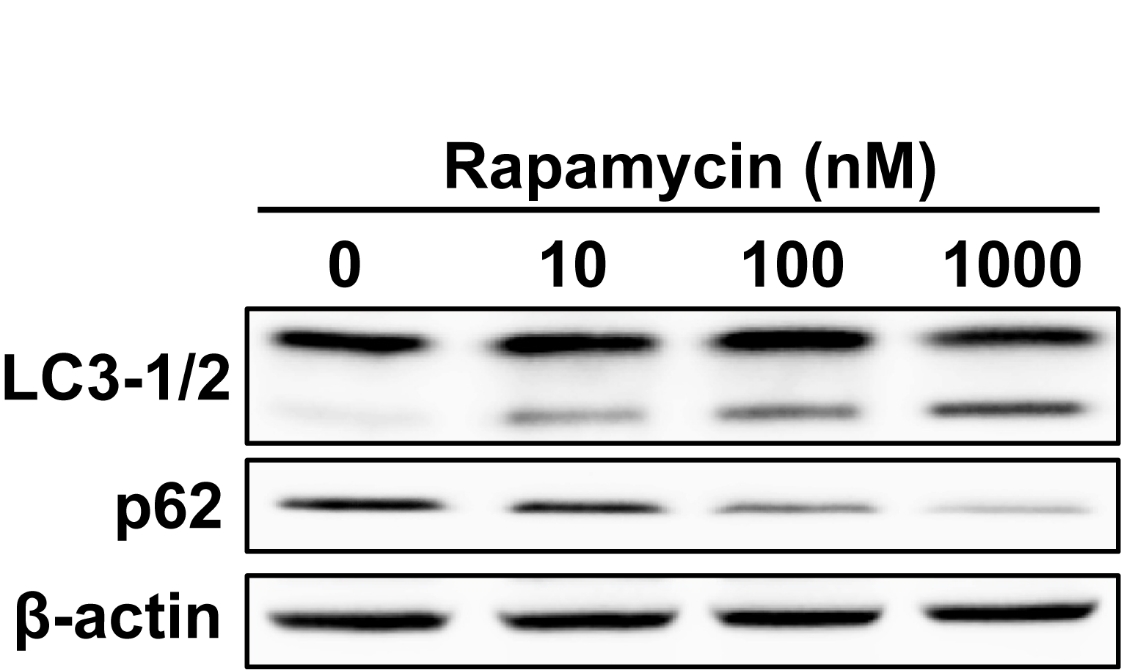
**

**Supplementary Fig. 2. Autophagy inducer rapamycin induced autophagic flux in A549 cells.** A549 cells were treated with various dose of rapamycin for 12 hours. After then LC3 and p62 was detected in total cell extracts by western blotting, using specific antibody. β-actin was used to assess the total amount of proteins loaded on the gel.

**
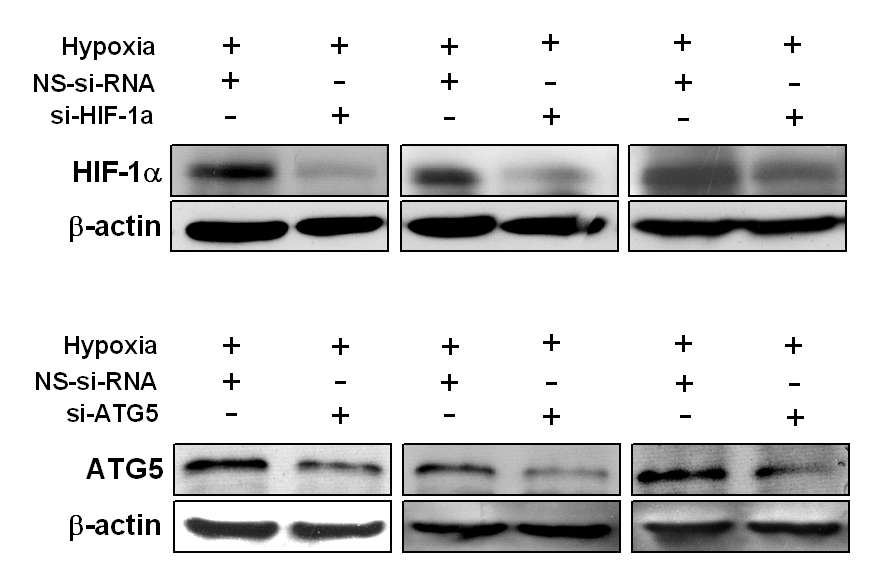
**

**Supplementary Fig. 3. Effect of HIF-1α, ATG5 silencing on HIF-1α, and ATG5 expression**. A 549 cells were transfected 24 h with anti-HIF-1α siRNA (siRNA) or anti-ATG5 siRNA or control siRNA. The transfection media were removed and replaced by culture media for 24 hours. Cells were then incubated under normoxic (N) or hypoxic (H) conditions for 12 hours. After transfection and incubation, HIF-1α and ATG5 was detected in total cell extracts by western blotting, using specific antibody. β-actin was used to assess the total amount of proteins loaded on the gel.


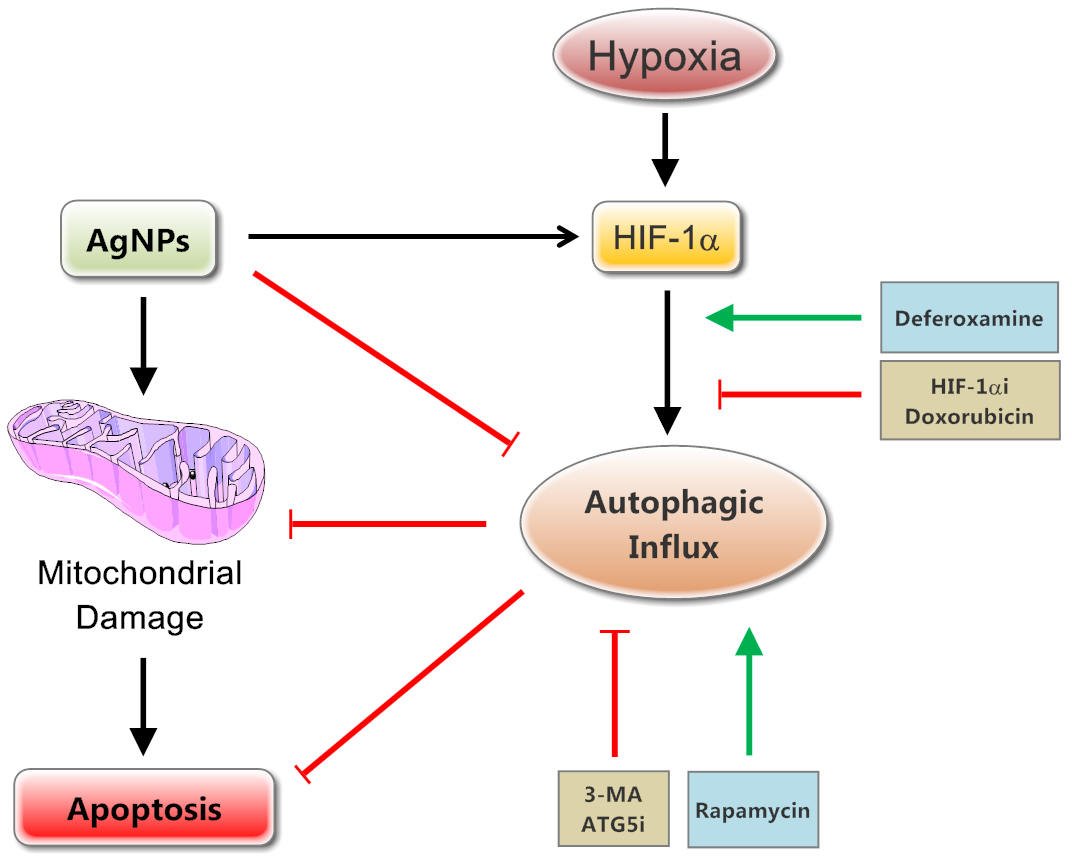


**Supplementary Fig. 4.** An underlying mechanism for explaining AgNPs-induced apoptosis. In lung cancer cells, a HIF-1α-mediated autophagy pathway modulates AgNPs-induced apoptosis
